# The effect of single and repeated doses of rivastigmine on gastric myoelectric activity in experimental pigs

**DOI:** 10.1371/journal.pone.0286386

**Published:** 2023-06-01

**Authors:** Chrysostomi Christina Tsianou, Jaroslav Kvetina, Vera Radochova, Darina Kohoutova, Stanislav Rejchrt, Martin Valis, Jana Zdarova Karasova, Ilja Tacheci, Veronika Knoblochova, Ondrej Soukup, Jan Bures

**Affiliations:** 1 Biomedical Research Centre, University Hospital, Hradec Kralove, Czech Republic; 2 Animal Laboratory, Faculty of Military Health Sciences, University of Defence, Hradec Kralove, Czech Republic; 3 The Royal Marsden NHS Foundation Trust, London, United Kingdom; 4 2nd Department of Internal Medicine—Gastroenterology, Faculty of Medicine in Hradec Kralove and University Hospital Hradec Kralove, Charles University, Hradec Kralove, Czech Republic; 5 Department of Neurology, Faculty of Medicine in Hradec Kralove and University Hospital Hradec Kralove, Charles University, Hradec Kralove, Czech Republic; 6 Department of Toxicology and Military Pharmacy, Faculty of Military Health Sciences, University of Defence, Hradec Kralove, Czech Republic; 7 Institute of Gastrointestinal Oncology, Military University Hospital Praha, Praha, Czech Republic; 8 Department of Medicine, First Faculty of Medicine, Charles University, Nové Město, Czech Republic; 9 Military University Hospital Praha, Praha, Czech Republic; Weizmann Institute of Science, ISRAEL

## Abstract

**Background:**

Rivastigmine is a pseudo-irreversible cholinesterase inhibitor used for therapy of Alzheimer’s disease and non-Alzheimer dementia syndromes. In humans, rivastigmine can cause significant gastrointestinal side effects that can limit its clinical use. The aim of this study was to assess the impact of rivastigmine on gastric motor function by means of electrogastrography (EGG) in experimental pigs.

**Methods:**

Six experimental adult female pigs (*Sus scrofa* f. *domestica*, hybrids of Czech White and Landrace breeds; 3-month-old; mean weight 30.7 ± 1.2 kg) were enrolled into the study twice and created two experimental groups. In group A, a single intragastric dose of 6 mg rivastigmine hydrogen tartate was administered in the morning to fasting pigs before EGG recording. In group B, rivastigmine was administered to overnight fasting animals in a dietary bolus in the morning for 7 days (6 mg per day). On day 8, an intragastric dose of 12 mg rivastigmine was given in the morning to fasting pigs before EGG. EGG recording was accomplished by means of an EGG standalone system. Recordings from both groups were evaluated in dominant frequency and EGG power (areas of amplitudes).

**Results:**

In total, 1,980 one-minute EGG intervals were evaluated. In group A, basal EGG power (median 1290.5; interquartile range 736.5–2330 μV^2^) was significantly higher in comparison with the power of intervals T6 (882; 577–1375; p = 0.001) and T10 (992.5; 385–2859; p = 0.032). In group B, the dominant frequency increased significantly from basal values (1.97 ± 1.57 cycles per minute) to intervals T9 (3.26 ± 2.16; p < 0.001) and T10 (2.14 ± 1.16; p = 0.012), respectively. In group B, basal EGG power (median 1030.5; interquartile range 549–5093) was significantly higher in comparison with the power of intervals T7 (692.5; 434–1476; p = 0.002) and T8 (799; 435–1463 μV^2^; p = 0.004).

**Conclusions:**

Both single as well as repeated intragastric administration of rivastigmine hydrogen tartrate caused a significant decrease of EGG power (areas of amplitudes) in experimental pigs. EGG power may serve as an indirect indicator of gastric motor competence. These findings might provide a possible explanation of rivastigmine-associated dyspepsia in humans.

## Introduction

**Fig 1:**
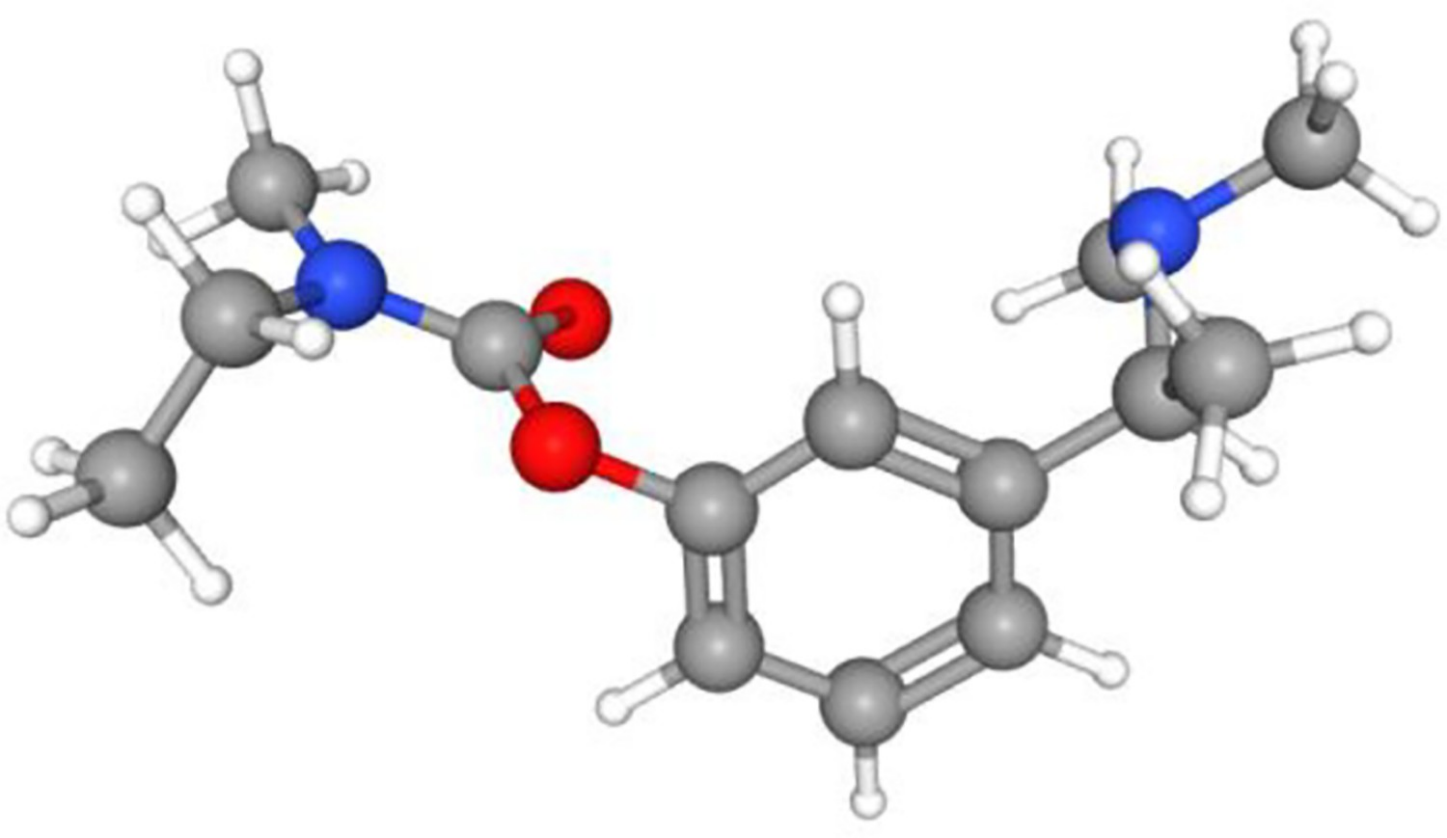
Structure of rivastigmine. 3D conformer [taken from ref. 2].

Rivastigmine is a pseudo-irreversible cholinesterase inhibitor used for therapy of Alzheimer’s disease and non-Alzheimer dementia syndromes (including vascular and Parkinson disease dementia) [1]. Rivastigmine (Fig 1 [ref. 2]) is a relatively weak (IC_50_ = 4.5 μM) but long-lasting (~ 10 hours) cholinesterase inhibitor that pseudo-irreversibly inhibits the action of both, acetylcholinesterase and butyrylcholinesterase. The exact mechanism of action has not yet been fully revealed in detail, but it is known that the rivastigmine mechanism of action occurs by inhibiting the hydrolytic activity of both enzymes by binding to their catalytic sites, thus resulting in a delay of acetylcholine breakdown in the synaptic cleft [3–7].

**Fig 2:**
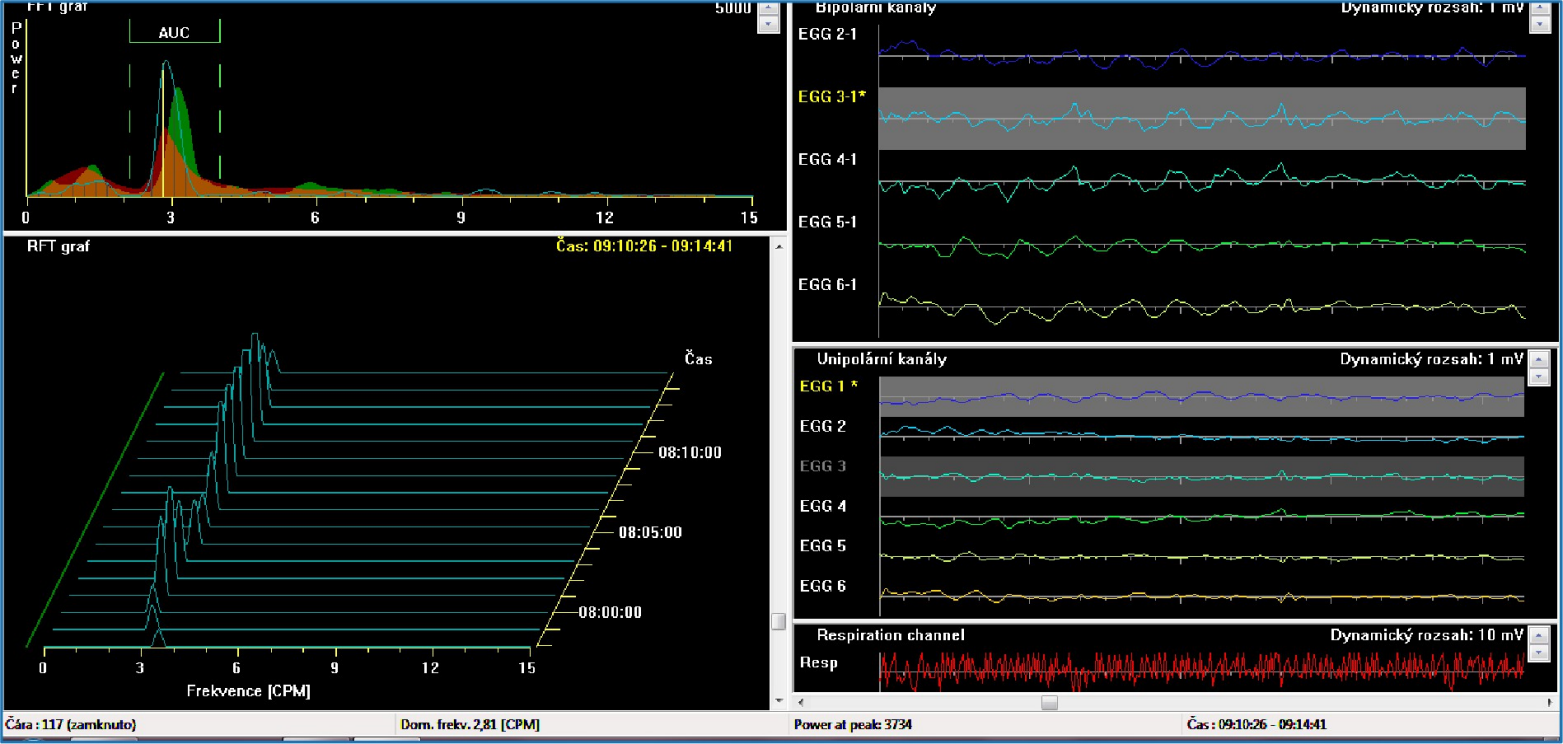
Normal basal porcine electrogastrography. Normal dominant frequency (3 cycles per minute)—lower left part of the picture. AUC: areas of amplitudes (EGG power)—upper left part of the picture; respiratory channel for recording breathing and movement artefacts as conveyed at the bottom right of the image (red line).

In humans, rivastigmine can cause significant gastrointestinal side effects, and hence its clinical use may be limited by these adverse effects. They include anorexia and weight loss (1–25% of treated patients), abdominal pain (~ 13%), dyspepsia and nausea (~ 17%), vomiting (~ 13%) and/or diarrhoea (~ 7%) [1, 3, 4, 8]. The mechanism of these adverse effects has not yet been fully clarified. Limited explanation is provided by the cholinergic effect of rivastigmine. In our previous work, we evaluated the effect of memantine [9] as well as the effect of acetylcholinesterase inhibitors (donepezil, galantamine) [10, 11] on gastric myoelectric activity in experimental pigs. To the best of our knowledge, there has been no literature concerning rivastigmine in experimental pigs published so far. However, based on studies with donepezil and galantamine [10, 11], we expect a similar effect of rivastigmine, i.e., class effect of acetylcholinesterase inhibitors, on gastric motor function in experimental pigs. Furthermore, similar findings in humans can be anticipated as the physiology of the gastrointestinal tract of pigs is similar to that of humans [12–14]. Taking this into account, porcine experimental models are suitable for preclinical studies and can be used for explanation of the adverse gastrointestinal effects of different drugs [15]. The aim of the current study was to assess the effect of a single as well as repeated doses of rivastigmine on gastric myoelectric activity in experimental pigs by means of electrogastrography (EGG). Normal human and porcine EGGs are fully comparable, Fig 2 [16–19].

## Materials and methods

### Animals

Six experimental adult female pigs (*Sus scrofa* f. *domestica*, hybrids of Czech White and Landrace breeds; 3-month-old; mean weight 30.7 ± 1.2 kg) were enrolled into group A. After a 14-day washout period, the same six pigs constituted group B. By the end of the experiment (four weeks from the beginning of the experiment) their mean weight was 36.0 ± 2.3 kg. The animals were purchased from a certified breeder (Stepanek, Dolni Redice, Czech Republic; SHR MUHO 2050/2008/41). The pigs were housed in an accredited animal laboratory (Faculty of Military Health Sciences, Hradec Kralove, Czech Republic). The pigs were fed with a standard assorted A1 food (Ryhos, Novy Rychnov, Czech Republic) with equal amounts twice a day, and had free access to drinking water.

### Design of the study

Six animals created two experimental groups. In group A, a single intragastric dose of 6 mg rivastigmine was administered in the morning to fasting pigs before EGG recording. In group B, rivastigmine was administered to overnight fasting animals in a dietary bolus in the morning for 7 days (6 mg per day). On day 8, an intragastric dose of 12 mg rivastigmine was given in the morning to fasting pigs before EGG recording. All intragastric administration of rivastigmine was carried out endoscopically using a video-gastroscope GIF-Q180 (Olympus Optical Co, Tokyo, Japan) dedicated for animal use only. Rivastigmine hydrogen tartate was purchased from Novartis, Praha, Czech Republic.

All EGG recordings were carried out under general anaesthesia. Intramuscular injections of ketamine (20 mg per kg; Spofa, Praha, Czech Republic) and azaperone (2.2 mg per kg; Janssen Animal Health, Saunderton, UK) were used as induction to the anaesthesia in all animals. Intravenous infusion of propofol (AstraZeneca AB, Stockholm, Sweden) was used for subsequent maintenance of general anaesthesia. Heart rate monitoring and pulse oximetry were used to secure the experiments.

### Electrogastrography

Our original method of porcine surface EGG which had been published before was used [8]. Briefly, EGG recording was accomplished by means of an EGG standalone system (MMS, Enschede, the Netherlands). Six active self-adhesive electrodes were placed on the upper part of the abdomen, and the 7th basal electrode was put to the left of the middle sternum. A special abdominal belt enabled identification of artefacts caused by breathing and body movements. Motion and breathing artefacts were removed automatically from the evaluation. A running spectral analysis was used for a standard evaluation of EGG. Results were conveyed as dominant frequency of gastric slow waves (cycles per minute) and EGG power (areas of amplitudes: μV^2).

### Statistical analysis

All data was tested statistically by means of the SigmaStat software (Version 3.1, Jandel Corp, Erkrath, Germany). Distribution of data was evaluated by Kolmogorov-Smirnov test, Shapiro-Wilko test was used for assessing the normality of sampled data. Descriptive statistics, unpaired t-test (for normal distribution) and Mann-Whitney rank sum test (for non-normal distribution) were used. Type 2 error beta was calculated when appropriate.

### Ethics

The Project was approved by the Institutional Review Board of Animal Care Committee of the University of Defence, Faculty of Military Health Services, Hradec Kralove, Czech Republic (protocol number MO 171673/2019-684800). Animals were held and treated in accordance with European Convention for the Protection of Vertebrate Animals [20].

## Results

In total, 1,980 one-minute high-quality EGG recordings were obtained (see Supporting Information file for detailed data). These one-minute recordings were aggregated into 15-minute intervals for final evaluation of the dominant frequency of gastric slow waves and EGG power (areas of amplitudes). Two outliers (0.1%) for dominant frequency and twelve outliers (0.6%) for power analysis from various time intervals of different animals in both groups were excluded from the final evaluation. An outlier is defined as a value outside the interval [*Q*_1_ – 1.5 *IQR*, *Q*_3_ + 1.5 *IQR*], where *Q*_1_ is lower quartile, *Q*_3_ is upper quartile and *IQR* = *Q*_3_ – *Q*_1_ is inter-quartile range.

**Fig 3:**
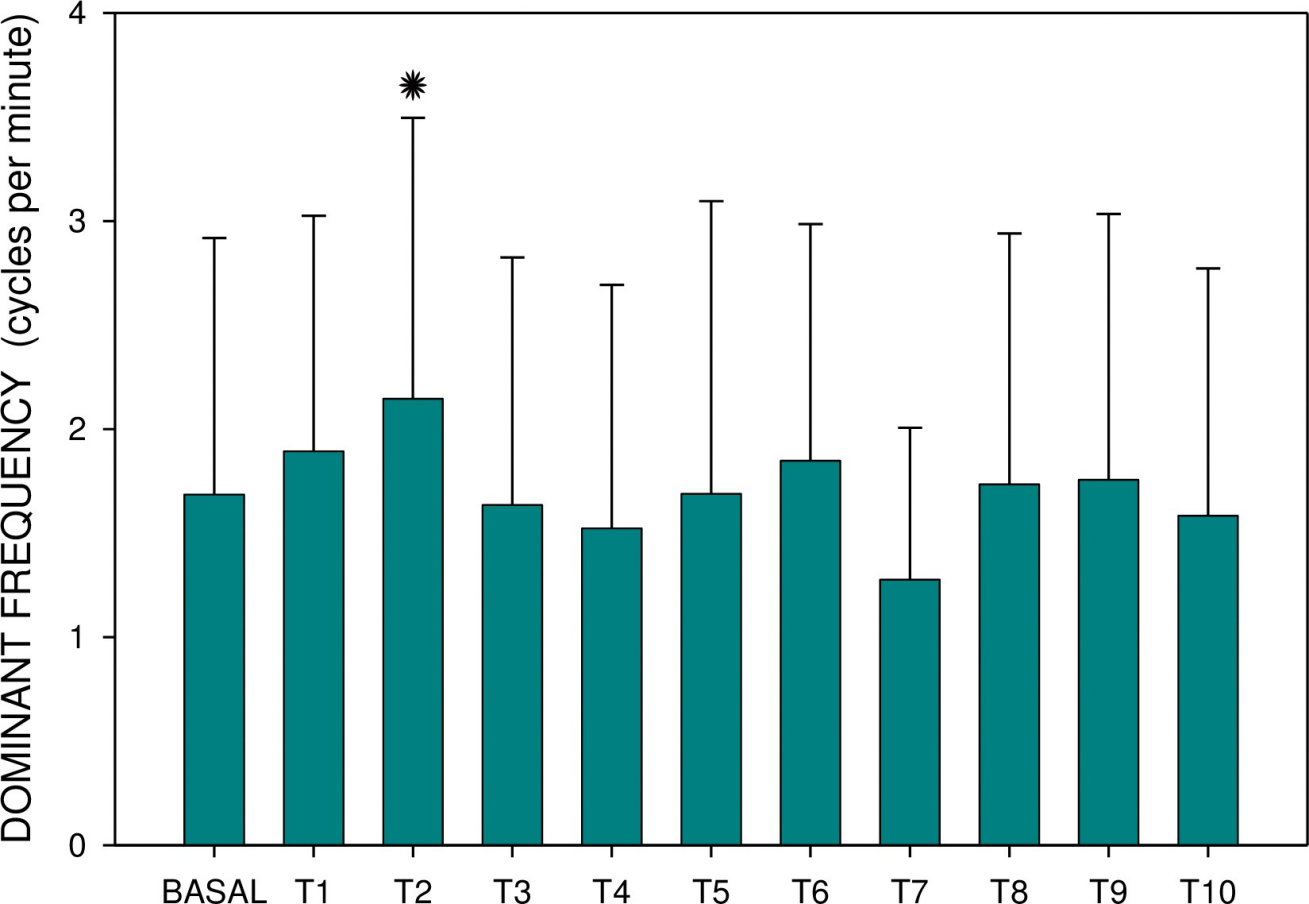
Electrogastrography. Group A: dominant frequency before and after a single intragastric administration of rivastigmine, 6 mg (mean + standard deviation). Outliers omitted. Note: BASAL: 15-minute basal recording before rivastigmine administration; T: 15-minute study recordings after rivastigmine administration (T1: time interval between 0–15 minutes. . . T10: time interval between 136–150 minutes). Asterisk indicates statistically significant difference in comparison to basal (p < 0.05).

**Fig 4:**
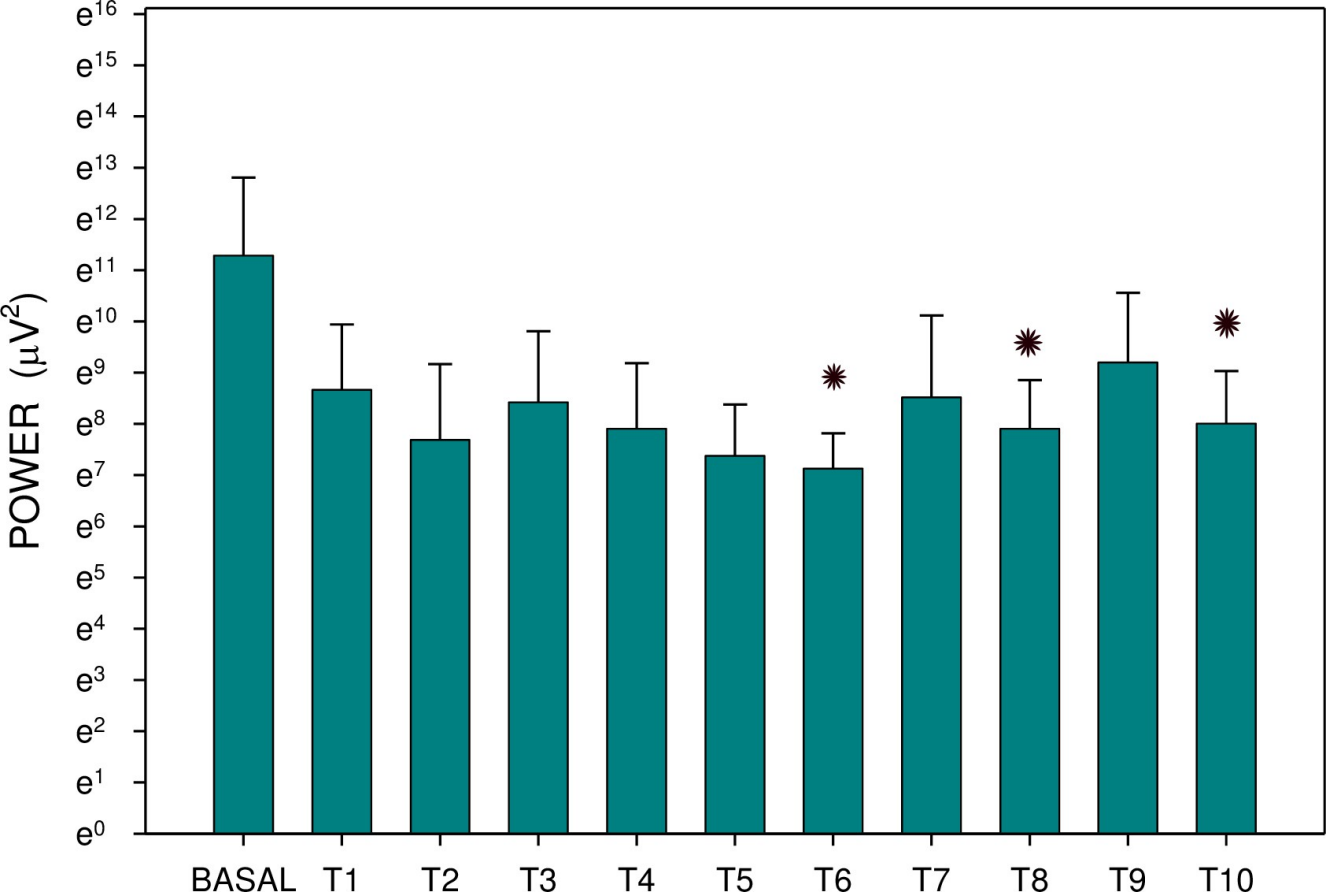
Electrogastrography. Group A: EGG power (areas of amplitudes) before and after a single intragastric administration of rivastigmine, 6 mg (mean + standard deviation). Outliers omitted. Y-Axis: natural logarithm scale. Note: BASAL: 15-minute basal recording before rivastigmine administration; T: 15-minute study recordings after rivastigmine administration (T1: time interval between 0–15 minutes. . . T10: time interval between 136–150 minutes). Asterisk indicates statistically significant difference in comparison to basal (p < 0.05).

**Fig 5:**
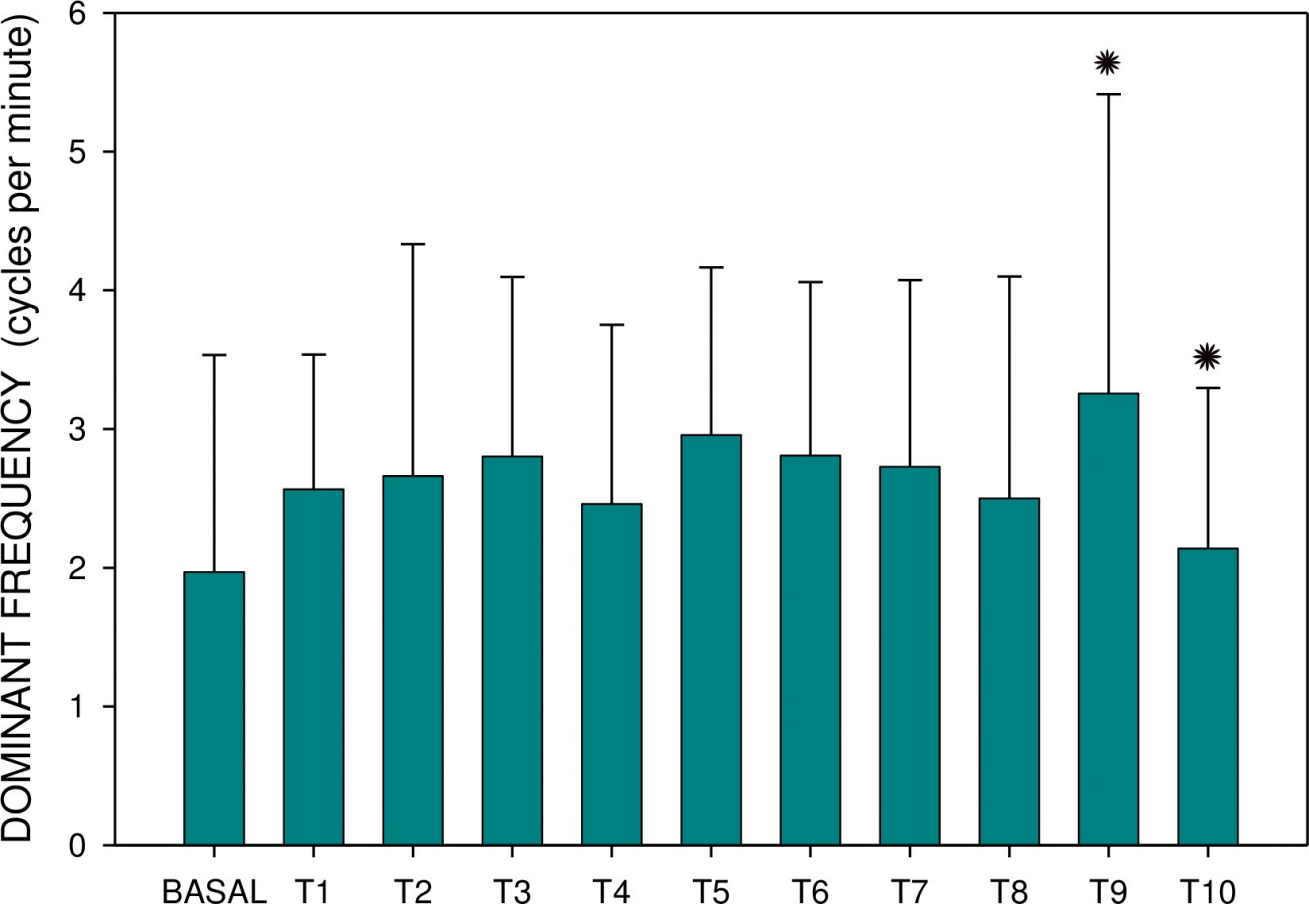
Electrogastrography. Group B: dominant frequency before and after the administration of an intragastric dose of rivastigmine, 12 mg, in animals with previous 7-day administration of rivastigmine, 6 mg daily (mean + standard deviation). Note: BASAL: 15-minute basal recording before rivastigmine administration; T: 15-minute study recordings after rivastigmine administration (T1: time interval between 0–15 minutes. . . T10: time interval between 136–150 minutes). Asterisk indicates statistically significant difference in comparison to basal (p < 0.05).

**Fig 6:**
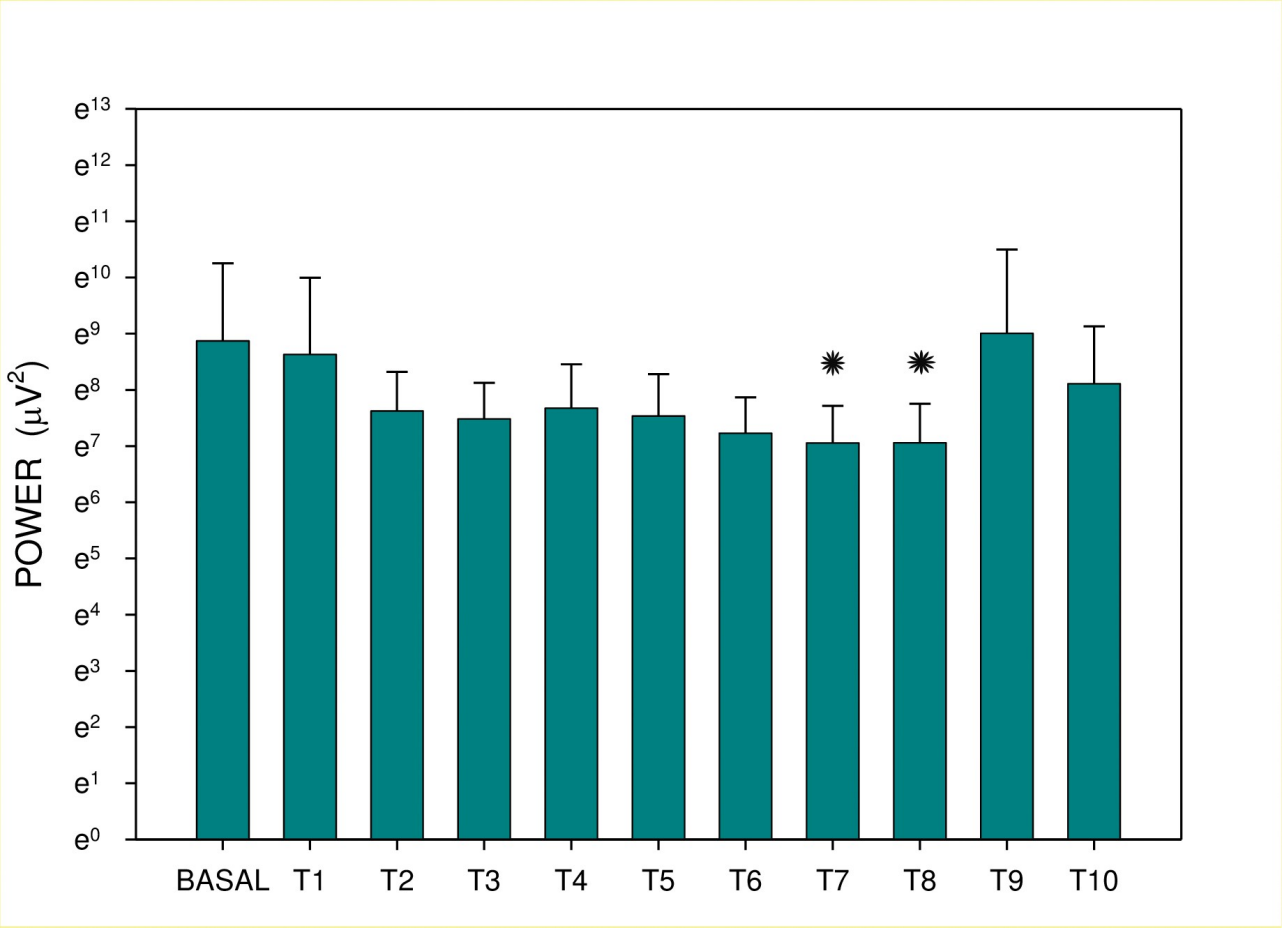
Electrogastrography. Group B: EGG power before and after the administration of an intragastric dose of rivastigmine, 12 mg, in animals with previous 7-day administration of rivastigmine, 6 mg daily (mean + standard deviation). Outliers omitted. Y-Axis: natural logarithm scale. Note: BASAL: 15-minute basal recording before rivastigmine administration; T: 15-minute study recordings after rivastigmine administration (T1: time interval between 0–15 minutes. . . T10: time interval between 136–150 minutes). Asterisk indicates statistically significant difference in comparison to basal (p < 0.05).

Major results are summarized in Figs 3–6. There was no significant difference between groups A and B either in basal dominant frequency (p = 0.399; type 2 error beta 0.862) or in basal EGG power (p = 0.539; type 2 error beta 0.416). In group A, the basal dominant frequency was significantly lower in comparison with the dominant frequency of interval T2 (p = 0.028); further differences were not statistically significant. Basal EGG power was significantly higher in comparison with power of T6 (p = 0.001) and T10 (p = 0.032). In group B, the dominant frequency increased significantly from basal values to T9 (p < 0.001) and T10 (p = 0.012), respectively. Basal EGG power was significantly higher in comparison with the power of intervals T7 (p = 0.002) and T8 (p = 0.004). Of note, non-significant trends (higher values of dominant frequency, decreased power) were observed in the latter intervals of EGG recording of group B.

## Discussion

In this experimental project, findings of significant importance regarding the impact of rivastigmine on the porcine gastric motor function were revealed. As far as we are aware, this is the first study on this topic. Thorough analysis of nearly two thousand one-minute EGG intervals has provided a detailed information of the impact of rivastigmine on porcine gastric motor activity. Based on previous studies of other acetylcholinesterase inhibitors [10, 11], similar effects of rivastigmine in humans and pigs could be expected.

The identical animals created two groups, all experimental pigs entered the current study twice. The first group simulated the very beginning of therapy with an initial single dose of rivastigmine, while the second one referred to long-term administration of the maintenance dose of this drug.

Although all changes of dominant frequency oscillated within normal ranges, clearly visible trends toward higher values (especially in the case of repeated administration of rivastigmine) could account for gastric myoelectric dysfunction. The most important finding is a clear trend and a significant decrease of the EGG power after both single as well as repeated administration of rivastigmine. At least in part, EGG power (areas of amplitudes) is an indirect indicator of gastric motor competence. These findings provide a possible explanation of rivastigmine-associated dyspepsia in the clinical setting. In humans, there is a correlation of EGG and gastric emptying rate estimated by ^13^C-octanoic acid breath test in healthy volunteers [21]. In patients with Roux-en-Y reconstruction after previous Billroth gastrectomy, an inverse trend between severity of dyspepsia and normal slow-wave rhythm percent activity in EGG was confirmed in the previous study [22]. In children with chronic intestinal pseudo-obstruction, the increased amplitude of the gastric electrical activity recorded by the EGG after a meal and erythromycin administration seemed to be only partly due to the increase in antral motor activity. The increase in power was also related to gastric distension in this study [23]. The effect on gastric myoelectrical activity of solely-administered erythromycin may enhance gastric motility and gastric emptying in patients with gastroparesis [24]. EGG and antro-duodenal manometry can complement each other in demonstrating gastric motor dysfunction in humans with functional dyspepsia [25].

Several mechanisms influence gastric myoelectric and motor control, both in humans and experimental setting, including the extrinsic nervous system, enteric nervous system, interstitial cells of Cajal, endocrine regulation, smooth muscles and immune-cell network [26]. Impact of many drugs has been a matter of diligent research, both in humans and experimental animals [27].

Different effect of neostigmine (increased) and atropine (decreased) was found in the direct and indirect recording of the gastrointestinal slow waves impulses in rats. Good correlation was found between maximal myoelectric power and smooth muscles contractions [28]. Non-invasive recording of gastrointestinal myoelectric activity was also used in experimental pigs to study different feedstuffs (standard formula and feedstuff with increased amount of fibre). Significant increase of the myoelectric power was found on the small intestinal pattern after a diet with increased amount of fibre [29]. Group of Robert Gáspár [30] used electromyography to record slow-waves myoelectric signals of the stomach, small intestine and large bowel to study stress-induced gastrointestinal dysmotility changes in awake rats. This study enabled to assess gastrointestinal changes as a consequence of dysregulation in the gut-brain axis. Diazepam and haloperidol were used, nevertheless, the method can be employed to investigate another drugs affecting the central nervous system through myoelectric response of the gastrointestinal tract [30]. Such an approach was used in humans, too. In healthy volunteers, similar alterations were found during a stress period in regards to gastrointestinal myoelectric activation as in the preclinical sample [31].

Despite all technical progress and advanced software support of current EGG equipments, the capability of spatial resolution is still missing [32, 33]. Future high-resolution electrical mapping will permit the recording and reconstruction of patterns of electrical activation in spatiotemporal detail [34–37]. However, routine clinical and experimental use has been still limited by the high cost of multichannel acquisition systems, difficulty in electrode construction, and the high complexity and time-intensiveness of analytical tasks [27].

In our experimental setting, we have found that similar decrease in EGG power was also revealed in pigs after the administration of other acetylcholinesterase inhibitors, i.e., donepezil [10] and galantamine [11]. The difference was more evident in animals with a longer intestinal transit time compared to those with a shorter intestinal transit time [11]. Notably, we used a single low dose of ketamine (an NMDA blocker) as induction to anaesthesia, so a possible gastric myoelectric effect of ketamine might have influenced the basal EGG recording. However, in our previous EGG study of memantine (another NMDA blocker), we did not find any significant effect of ketamine [9]. Another important consideration relates to the gender difference of motor gastrointestinal function both in humans and experimental pigs, as we have previously investigated in experimental pigs [38]. Therefore, our current study was carried out on adult female pigs only.

Translation impact may be limited by the fact that there is an important difference between healthy humans and patients suffering from Alzheimer’s disease. Peak activity of rivastigmine is reached more slowly in those with Alzheimer’s disease compared to healthy subjects, and the inhibitory effects induced by rivastigmine have a longer duration (6 vs 2.5 hours and 12 vs 8.5 hours, respectively). In contrast to other acetylcholinesterase inhibitors, the hepatic cytochrome P-450 system is not involved in the metabolism of rivastigmine [39–42]. On the other hand, our current study demonstrated the direct impact of rivastigmine on porcine myoelectric activity, which may well be of a significant clinical importance. Pathological EGG is an indirect marker of gastric motor disorders in humans which are associated with dyspepsia, nausea and vomiting. Potential practical impact can be the awareness of such a mechanism followed by an impulse for similar studies in Alzheimer’s disease.

We are aware of possible limitations of our current experimental study. We did not investigate any biochemical / pharmacokinetic parameters. Furthermore, we did not titrate body-weight-based doses of rivastigmine. In humans, recommended doses are the highest ones which are still well tolerated long-term, regardless of the body weight: initial dose 3 mg per day, afterwards gradually increased up to a maintenance dose (12 mg per day) in humans. A single oral dose of 1.0 mg/kg was used in experimental pigs previously [43]. Chosen intragastric doses in our experimental study were therefore comparable with the clinical setting. According to our previous experience, the impact of cholinergic and anticholinergic agents on porcine gastric motor activity was dose dependent [19, 44]. However, an indisputable advantage of our current project is the fact that both groups consisted of the identical animals. High variability is an essential, well-known characteristic of all gastric electrophysiology, both in humans and experimental pigs, taking in the consideration normal rhythm of three cycles per minute. So that each one-minute recording is a moving average of three subsequent periods, thus eliminating risks of bias and confounding.

## Conclusions

Both, single as well as repeated intragastric administration of rivastigmine hydrogen tartrate caused an obvious tendency to decrease of the EGG power (areas of amplitudes) in experimental pigs. EGG power may serve as an indirect indicator of gastric motor competence. These findings might provide a possible explanation of rivastigmine-associated dyspepsia in humans, however, further experiments are needed to confirm our findings.

## Supporting information

S1 File (PDF)
