# Correction: The effect of single and repeated doses of rivastigmine on gastric myoelectric activity in experimental pigs

**DOI:** 10.1371/journal.pone.0315834

**Published:** 2024-12-11

**Authors:** Chrysostomi Christina Tsianou, Jaroslav Kvetina, Vera Radochova, Darina Kohoutova, Stanislav Rejchrt, Martin Valis, Jana Zdarova Karasova, Ilja Tacheci, Veronika Knoblochova, Ondrej Soukup, Jan Bures

There are errors in the author affiliations. The correct affiliations are as follows:

Chrysostomi Christina Tsianou^1^, Jaroslav Kvetina^1^, Vera Radochova^2^, Darina Kohoutova^1,3^, Stanislav Rejchrt^4^, Martin Valis^5^, Jana Zdarova Karasova^1,6^, Ilja Tacheci^4^, Veronika Knoblochova^1^, Ondrej Soukup^1^, Jan Bures^1,7,8^

**1** Biomedical Research Centre, University Hospital Hradec Kralove, Czech Republic, **2** Animal Laboratory, University of Defence, Faculty of Military Health Sciences, Hradec Kralove, Czech Republic, **3** The Royal Marsden NHS Foundation Trust, London, United Kingdom, **4** 2nd Department of Internal Medicine—Gastroenterology, Charles University, Faculty of Medicine in Hradec Kralove and University Hospital Hradec Kralove, Czech Republic, **5** Department of Neurology, Charles University, Faculty of Medicine in Hradec Kralove and University Hospital Hradec Kralove, Czech Republic, **6** Department of Toxicology and Military Pharmacy, University of Defence, Faculty of Military Health Sciences, Hradec Kralove, Czech Republic, **7** Institute of Gastrointestinal Oncology, Military University Hospital Prague, Czech Republic, **8** Department of Medicine, Charles University, First Faculty of Medicine and Military University Hospital Prague, Czech Republic.
