# Supplementary material for: The effect of single and repeated doses of rivastigmine on gastric myoelectric activity in experimental pigs
Source: PLoS One. 2023 Jun 1;18(6):e0286386. doi: 10.1371/journal.pone.0286386 (PMC10234519; doi:10.1371/journal.pone.0286386)
Supplement: S1 File — (PDF) [file pone.0286386.s001.pdf]

	Animal No.	Bas-riv-6-df	Bas-riv-6-pow	riv-6-df T1
Rivastigmin 6 mg	311 a	1,17	6477	3,05
		3,05	5701	3,05
		3,05	4371	3,05
		0,94	1763	0,94
		0,47	1299	1,41
		0,47	833	1,41
		1,17	1596	0,94
		0,94	2315	1,64
		1,17	1080	1,64
		1,17	522	0,94
		1,17	252	1,17
		1,64	209	1,17
		3,28	204	1,41
		3,05	527	1,64
		3,05	718	0,7
	312 a	1,41	244	0,7
		1,41	489	0,7
		1,41	874	3,28
		1,17	1063	3,28
		1,17	601	0,47
		2,11	376	3,28
		3,28	297	3,52
		1,17	755	3,52
		1,17	1576	3,28
		3,28	1276	0,7
		3,28	597	0,7
		1,41	409	1,17
		3,28	1273	1,17
		0,7	4687	0,94
		0,7	6913	0,94
	313 a	0,47	4003086	1,17
		0,47	3192364	1,17
		1,17	4268831	0,47
		1,17	3834469	0,94
		1,41	1154163	0,94
		0,94	1859982	0,94
		0,94	3607019	0,47
		0,94	2227405	0,94
		0,7	925287	2,81
		0,47	635664	2,81
		0,7	473456	2,58
		1,17	218628	1,64
		0,7	233928	2,58
		0,47	127280	2,58
		1,17	938104	1,17
	314 a	0,7	1807	3,28
		0,7	2604	3,28
		0,7	1470	0,94
		4,69	1127	3,28

	4,69	1307	3,28
	3,98	1372	3,52
	4,92	1282	5,39
	4,45	1597	3,75
	4,45	1831	0,47
	4,45	1181	0,47
	3,75	1169	1,17
	3,75	713	1,17
	1,17	794	1,17
	1,17	713	3,75
	3,28	1407	3,75
315 a	0,94	7078	2,34
	1,17	2191	2,34
	3,05	1204	2,34
	3,05	1210	2,34
	3,05	769	0,7
	3,05	271	0,7
	1,17	582	0,7
	0,7	440	2,58
	1,17	759	2,58
	0,94	1992	2,58
	0,7	5102	2,81
	0,7	4589	2,81
	0,7	2150	1,17
	2,34	5788	2,81
	2,34	13239	2,81
316 a	0,7	2048	0,94
	0,47	1200	2,81
	1,17	904	2,81
	0,94	1023	1,17
	0,94	2345	3,05
	0,94	1568	3,28
	0,94	457	1,17
	1,64	690	0,94
	1,17	1807	1,17
	0,94	1475	0,94
	0,47	1083	0,94
	0,94	1507	0,7
	0,94	1817	0,94
	0,94	1070	0,47
	0,94	596	3,75

riv-6-pow T1	riv-6-df T2	riv-6-pow T2	riv-6-df T3	riv-6-pow T3	riv-6-df T4	riv-6-pow T4	riv-6-df T5
718	0,7	438	1,17	845	0,94	587	0,7
701	0,47	148	1,17	464	0,94	429	0,94
430	0,94	130	0,47	298	0,94	290	1,41
401	0,7	181	0,94	285	0,94	157	1,64
490	0,7	215	0,94	546	0,7	353	1,41
424	0,94	269	0,94	401	0,7	536	1,41
292	0,94	672	0,7	196	0,94	501	0,47
417	0,7	443	3,98	92	0,94	260	0,47
834	1,41	168	1,17	167	1,17	378	0,47
2984	1,64	308	0,7	307	1,17	801	0,47
18023	0,94	900	0,7	137	1,17	904	0,47
11519	0,94	1450	3,28	120	1,17	694	1,17
1139	0,94	1114	1,41	83	0,94	312	1,17
375	0,94	666	1,17	276	0,7	227	1,17
468	1,41	789	0,94	719	0,7	553	0,94
6913	0,94	62117	1,41	2625	0,94	2554	0,47
3244	0,94	34416	1,41	1085	0,94	4797	2,81
3220	0,7	5637	1,17	1211	0,94	28305	0,94
2144	3,05	1042	1,41	1933	0,94	57394	3,75
1639	3,28	747	1,64	4786	0,94	15650	1,17
1781	3,28	1506	0,23	3684	1,17	1048	1,17
1345	3,52	746	0,94	3269	1,17	461	1,17
1479	3,05	1187	0,94	2895	0,7	800	2,81
1725	3,28	3883	0,94	4079	0,7	888	0,94
3344	3,28	3143	0,94	7808	3,05	595	0,94
3781	0,94	3365	1,17	4860	2,81	629	0,7
1207	0,94	2773	1,17	3217	0,94	701	3,28
2714	3,28	2492	1,17	1411	1,17	539	3,28
7623	3,05	3598	3,28	1655	2,58	270	3,05
26152	1,41	4854	3,28	1215	0,47	618	2,81
938104	2,58	955	0,94	1188	0,7	1044	0,7
2251112	2,58	1784	0,7	2163	1,17	2478	0,94
1821147	1,17	2985	0,94	45958	0,94	8385	1,17
2087916	2,58	1559	0,94	116217	1,41	4643	1,41
1942518	2,58	1486	0,94	31534	1,64	1344	0,7
420741	0,94	1669	1,17	2868	0,47	833	0,7
20073	0,7	1273	1,41	443	0,7	3364	0,7
5406	2,81	928	1,41	1168	0,7	2802	1,17
3038	1,41	692	1,41	2294	0,7	1211	1,17
3077	0,94	995	0,47	1701	0,7	743	0,94
2642	0,7	1127	0,47	2476	1,41	1412	0,94
1088	3,05	499	0,94	1985	1,41	4114	1,41
1415	3,05	1266	1,17	4630	1,41	4234	1,41
1459	3,05	1427	0,94	5292	0,7	5385	1,41
799	0,94	1687	0,7	3020	0,7	5073	0,7
1407	3,75	631	3,98	529	0,94	558	4,22
1578	3,75	626	3,98	313	0,7	1121	4,22
1173	3,75	581	0,47	786	0,7	1013	4,22
830	3,98	802	1,17	3755	3,28	747	4,22

899	3,98	851	1,17	2839	3,28	1892	8,2
749	3,75	547	1,88	911	4,22	1869	8,2
900	1,17	352	3,98	1002	4,22	1344	1,41
452	1,17	418	3,98	1582	4,22	731	1,41
358	3,75	541	3,98	1815	4,22	942	0,94
335	3,75	622	3,98	1654	4,22	903	0,94
850	3,28	249	3,98	1050	4,22	895	0,94
1344	8,2	177	3,98	890	1,17	1456	0,94
571	3,98	322	0,94	1761	1,17	958	0,94
868	3,98	428	3,98	2568	4,22	744	4,45
1248	3,98	457	3,98	1765	4,22	1275	4,22
13239	0,7	3066	1,17	12716	0,94	3431	1,64
19882	0,7	1864	1,17	25441	0,94	1831	1,64
15078	3,05	1229	0,94	22145	0,94	1296	1,41
5772	0,94	1538	1,17	3041	1,17	903	1,41
40451	3,05	2599	1,17	5258	0,94	913	1,41
119830	2,81	1414	1,41	9276	0,94	1301	1,41
46804	0,94	955	1,64	3100	1,17	2280	1,41
9273	0,7	625	0,7	2064	0,94	1628	1,88
7722	2,81	549	0,94	1840	3,98	1105	2,11
9058	1,17	975	1,41	1099	0,94	3790	1,41
11142	2,81	953	3,98	805	0,94	15549	1,41
4050	2,81	993	3,98	798	0,7	11828	1,64
762	3,05	852	3,98	577	0,7	2655	1,64
1223	3,05	905	0,94	1051	3,98	743	1,41
2087	1,17	3185	0,94	3070	1,41	1587	1,41
596	3,75	287	1,17	603	0,7	419	1,17
766	3,75	370	0,94	4212	4,22	182	0,47
508	0,7	585	0,94	5244	3,98	290	0,7
570	0,7	712	0,7	1039	1,88	612	0,7
744	1,41	648	1,41	475	1,64	322	1,64
566	0,7	167	1,64	1424	0,7	332	1,41
350	0,7	487	0,94	810	0,7	736	1,41
581	1,64	500	1,17	907	0,7	876	1,17
1011	3,75	359	1,17	814	0,94	885	1,17
4164	1,41	269	0,94	909	1,64	736	0,94
7606	2,81	266	0,94	1170	1,64	352	3,75
2253	1,88	661	0,7	483	0,94	279	1,41
211	1,88	916	3,28	232	1,17	295	1,64
139	1,88	868	4,22	218	1,17	496	1,64
177	1,88	742	0,7	521	1,17	486	0,94

riv-6-pow T5	riv-6-df T6	riv-6-pow T6	riv-6-df T7	riv-6-pow T7	riv-6-df T8	riv-6-pow T8	riv-6-df T9
524	0,7	203	0,7	354	0,7	696	0,94
265	0,7	489	0,7	159	0,94	289	0,94
133	0,94	1309	0,94	112	0,94	301	1,17
182	0,94	883	1,17	185	0,94	244	1,64
321	0,94	193	0,94	154	1,41	404	1,41
492	1,41	329	1,17	232	1,41	436	1,41
332	1,17	527	1,17	555	1,41	302	0,7
441	1,17	617	0,47	372	0,94	503	0,7
314	0,94	764	0,47	354	0,94	373	1,64
175	1,17	403	0,94	309	1,64	354	1,64
173	0,7	421	1,17	299	1,64	265	1,88
229	0,94	644	1,41	182	0,94	280	1,64
198	0,94	545	0,94	284	1,17	393	1,17
113	0,94	472	0,7	253	1,17	769	0,94
143	0,7	320	0,7	498	0,94	580	1,41
411	1,41	2087	3,52	900	0,94	2182	3,05
236	3,28	1783	3,52	1276	0,94	4544	0,7
314	3,28	2069	0,94	791	0,7	4543	1,17
334	3,28	2002	2,81	1070	0,94	1248	1,41
923	3,28	3796	2,81	859	0,94	821	3,28
3435	3,05	3736	0,47	790	1,17	1373	3,05
1541	3,05	2869	0,94	1629	1,17	1457	2,81
864	2,81	580	1,17	2583	1,17	1146	0,7
887	2,81	359	1,17	1587	1,17	1318	3,28
2394	2,81	809	0,94	856	1,41	886	3,28
1350	2,81	659	0,7	1230	1,17	725	1,41
712	3,28	1157	0,7	1877	1,17	2161	1,41
949	3,28	1013	0,7	1264	1,17	2370	1,41
1209	2,81	840	2,81	711	0,7	1028	1,41
948	2,81	1171	2,58	742	3,28	396	2,81
4039	1,17	1159	1,41	29679	0,94	1522	0,47
2222	0,47	992	1,17	38258	3,05	627	0,47
1446	0,7	1375	1,17	9953	1,41	1159	0,47
850	0,94	498	1,17	2784	1,41	3006	0,47
4059	3,05	637	1,17	5139	1,17	10566	0,47
16909	2,11	951	1,17	3977	0,7	31731	0,94
17917	2,11	619	1,41	2076	0,47	15131	0,94
10066	1,17	711	1,41	1956	0,47	2524	0,94
3602	1,17	891	1,17	17568	0,94	3130	1,17
3531	1,17	955	0,94	144071	0,47	3976	1,17
1139	1,17	1163	0,94	113994	0,47	2730	0,94
812	1,41	1148	1,17	7505	1,41	1578	0,7
1751	1,41	1707	1,17	1463	1,17	2126	0,7
2157	1,17	3095	1,17	1382	0,7	2075	0,7
2369	1,41	8679	1,17	1907	0,7	6908	0,94
1516	3,52	699	3,75	3610	3,98	3081	4,22
1242	3,52	663	3,98	2333	3,98	5885	4,22
969	3,98	640	0,7	1128	3,98	7307	4,22
671	12,89	608	0,94	1899	3,98	6427	4,22

407	12,89	1395	0,94	971	3,98	5469	4,22
293	3,98	909	1,17	613	3,98	6963	4,22
755	14,06	1435	1,17	1035	4,22	7412	4,22
866	0,94	920	1,17	1404	4,22	8749	4,22
1141	0,94	751	0,94	1158	4,22	7627	4,22
1612	2,58	462	0,94	807	4,22	7073	4,22
2310	3,98	548	0,7	422	4,22	6055	4,22
3260	3,98	1267	1,17	370	4,22	6813	4,22
2358	3,98	2595	0,94	1089	4,22	9071	4,22
1276	3,98	2543	0,94	1764	3,98	7228	4,22
1898	3,98	2245	1,17	1458	4,22	7423	4,22
2245	0,94	582	0,94	3770	2,11	306	0,7
1308	1,17	794	1,17	1603	1,41	1076	0,7
708	1,41	1084	1,88	1694	1,17	3097	0,7
1853	1,17	881	0,7	943	1,17	1742	0,7
2215	0,94	1101	1,41	776	0,47	677	1,41
1288	1,64	876	1,64	1572	1,64	505	1,17
1177	1,41	1573	1,88	1447	1,41	880	1,17
1394	1,41	2142	0,47	1150	1,17	1140	1,17
986	1,41	1845	1,88	860	2,58	978	1,41
1533	1,41	1375	2,11	1054	2,58	1293	1,41
3146	1,41	1291	1,17	921	1,17	493	0,7
1978	1,17	2009	1,41	693	1,88	452	1,41
926	0,94	5041	1,41	389	3,05	446	1,41
1674	1,17	4493	2,34	280	3,05	643	1,41
715	0,94	3014	2,34	542	0,94	1432	1,41
159	0,7	531	1,17	169	0,7	227	0,94
294	0,7	918	0,7	806	1,41	201	0,7
777	0,94	1137	0,7	1572	1,64	95	1,17
657	0,94	926	0,94	980	0,7	610	0,94
565	0,94	587	1,17	748	0,7	1361	1,41
538	0,94	446	1,17	607	0,7	764	1,41
543	0,94	544	0,94	194	0,94	227	1,17
932	1,17	577	0,94	399	1,41	225	1,17
652	1,17	777	1,17	679	1,41	172	0,7
204	1,17	748	0,94	480	1,88	173	1,17
168	1,17	357	0,7	328	1,64	447	0,94
390	1,17	205	0,47	426	1,41	302	0,7
412	3,98	475	0,47	406	1,41	129	0,7
319	3,98	430	1,41	237	1,17	124	2,34
216	3,98	168	1,41	174	0,94	335	0,47

riv-6-pow T9	riv-6-df T10	riv-6-pow T10	riv-6-df T11	riv-6-pow T11	riv-6-df T12	riv-6-pow T12
504	1,41	391	1,17	160	1,88	71
542	1,17	404	1,17	522	0,94	68
225	1,17	235	0,94	625	1,41	119
238	0,94	214	0,94	1074	1,41	129
348	1,17	212	0,94	1589	1,17	110
146	1,17	389	1,17	522	1,88	86
518	1,17	442	1,17	215	1,64	324
722	0,94	207	1,41	219	0,94	409
363	1,41	186	1,64	207	0,94	265
185	1,41	163	1,64	575	3,05	198
347	1,41	139	1,41	1020	3,28	157
259	0,7	393	1,41	478	0,7	170
341	0,7	1048	0,7	102	1,64	392
240	0,7	644	0,7	124	1,64	467
290	0,7	161	1,41	124	1,41	399
427	0,7	789	1,17	482	1,88	548
302	0,7	973	1,64	290	0,7	1986
436	0,7	580	0,94	612	0,94	10059
499	1,17	371	0,94	2808	1,17	19374
317	0,94	1252	0,94	3334	1,17	13924
299	0,94	915	3,52	804	0,94	6754
339	1,17	439	3,52	505	0,94	5927
158	1,88	171	0,7	235	0,94	3429
187	1,41	385	0,7	266	1,17	7110
381	1,41	696	0,7	649	0,94	5570
328	1,41	694	0,94	454	0,94	1818
697	1,41	523	1,41	469	0,94	4239
883	1,64	496	1,17	399	0,94	6483
604	1,64	637	3,52	197	0,94	1942
492	0,94	551	1,41	176	3,05	246
8097	0,94	31784	0,7	1466	0,94	2894
15672	1,17	19088	0,7	706	1,17	720
64281	1,41	1793	1,17	2869	1,17	1065
117350	1,41	1012	0,47	3908	1,17	1303
56112	0,7	2805	0,7	3544	1,17	1834
11331	0,7	2182	0,94	2692	1,41	1272
8325	0,94	7172	1,17	2953	0,7	1479
6475	0,94	25582	1,17	2651	1,41	1090
10891	0,7	11792	1,17	2838	1,41	800
97827	0,7	7969	1,17	5901	1,17	977
208993	0,94	14300	1,17	4914	0,47	829
68999	0,7	8745	1,17	1642	0,7	1401
11572	0,7	10491	0,94	4287	0,7	1586
11572	0,94	17768	0,94	19086	1,17	994
8317	0,94	7414	0,94	15500	1,17	1219
9084	4,22	4584	0,7	472	4,22	859
7761	4,22	4962	1,41	457	3,98	1977
7455	4,22	6377	1,64	407	4,22	2593
7261	4,22	6978	12,42	936	4,22	2316

7969	4,22	5809	1,41	1389	4,22	1709
9222	4,22	3901	0,7	622	4,22	1851
9013	3,98	3997	1,41	521	4,22	2547
9943	3,98	3972	1,41	977	4,22	3975
11400	3,98	3187	3,75	1141	1,17	8299
12576	3,98	2746	3,98	1268	1,17	9554
14041	3,98	1628	3,98	1888	0,94	9961
15449	3,98	1077	3,98	2183	4,22	11236
14168	3,98	1731	3,98	1436	4,22	13593
8704	3,98	1860	4,22	540	4,22	13969
5323	3,98	1014	0,7	642	4,22	9671
869	1,41	2899	1,17	3169	1,41	2884
684	1,64	1753	1,17	1887	1,41	2944
964	0,7	2076	1,17	2206	0,94	6158
539	0,7	2390	0,94	5799	0,94	9597
457	0,47	1876	0,94	5358	0,94	3627
677	0,47	3020	0,94	1909	1,41	5147
1817	0,47	2859	0,94	567	1,41	4313
2869	0,7	1979	0,94	802	1,41	2710
2266	0,7	675	0,94	1657	3,75	2621
1307	0,94	1048	0,94	2842	1,17	6508
819	0,94	2416	1,17	3722	1,41	8439
360	1,41	1242	1,17	3626	1,41	4784
404	1,64	1281	1,17	2860	1,41	1520
1176	1,64	760	1,17	3541	1,17	1280
2871	1,17	1583	0,47	3540	1,41	1221
574	0,47	283	0,7	363	0,7	407
641	0,7	323	0,47	205	0,7	173
373	1,41	129	0,94	315	0,94	109
467	1,41	137	0,94	593	1,17	56
286	1,41	414	0,94	300	0,7	136
317	1,41	303	1,17	77	0,94	308
412	0,7	130	1,41	61	0,94	527
209	0,7	117	1,41	142	0,94	516
189	1,41	106	1,64	214	3,05	394
92	3,28	219	1,64	202	3,05	99
146	1,17	215	0,7	159	1,41	185
239	1,17	117	0,7	199	1,17	420
190	1,17	275	3,28	204	0,7	421
117	1,17	550	0,94	165	0,7	733
142	0,7	549	0,7	375	0,7	546

riv-6-df T13	riv-6-pow T13	riv-6-df T14	riv-6-pow T14	riv-6-df T15	riv-6-pow T15	riv-6-df T16
1,17	377	1,41	502	1,64	706	0,7
1,64	237	0,94	669	3,52	430	0,94
1,88	251	1,64	360	1,64	715	2,34
1,88	177	1,41	446	1,64	869	1,17
1,17	298	0,94	870	1,64	645	1,17
1,17	228	0,94	756	1,41	658	1,17
1,17	279	1,41	805	1,41	513	0,94
1,17	511	1,41	549	1,64	379	1,17
1,41	468	1,17	491	1,17	364	0,7
0,47	308	1,17	1043	1,41	815	0,7
2,34	325	1,17	858	1,17	1310	1,88
1,17	440	1,64	381	0,94	910	2,11
1,17	685	0,7	361	1,41	704	1,41
1,17	621	0,94	613	1,17	572	1,17
1,17	325	1,88	1037	1,17	509	1,17
3,05	264	1,88	1728	14,06	732	3,98
3,75	326	14,77	1002	3,98	882	13,13
1,17	823	14,53	1794	0,94	2324	12,89
1,17	1607	3,98	1793	0,94	3788	0,94
1,17	2486	0,94	2999	3,98	1703	0,94
1,17	1368	0,94	4248	4,22	1108	3,98
3,75	556	3,98	2263	13,83	817	1,17
14,53	819	1,41	2946	0,94	2744	1,17
3,98	1320	0,94	3170	0,94	4331	1,17
3,98	1671	0,94	2595	0,94	3390	1,17
1,17	2061	0,94	1645	1,17	1695	1,17
1,41	5086	0,94	796	1,17	4892	14,06
1,41	4672	0,94	849	1,17	3259	13,13
1,41	3697	1,17	433	1,41	475	13,13
1,88	2848	14,3	381	14,77	619	13,13
0,94	2349	0,94	1650	0,94	2289	1,17
0,94	3828	1,17	1368	0,94	1640	1,17
0,94	2518	1,17	1647	0,94	1930	1,41
0,94	539	1,17	2196	0,7	5064	1,17
0,7	303	1,41	3413	0,94	4405	0,94
1,41	415	1,41	1778	1,64	2575	0,94
1,41	814	0,94	2471	1,41	3455	0,94
1,41	952	0,94	3680	0,7	3727	0,94
1,41	1640	0,94	1245	0,7	3098	0,94
1,41	2357	1,41	6054	0,7	2641	1,17
1,17	2177	1,17	4172	0,7	2095	1,17
0,94	2310	0,94	1298	0,94	1111	0,94
0,94	7251	0,7	910	0,94	948	0,7
0,94	5563	0,94	778	0,47	739	0,7
0,94	2019	0,94	1774	3,05	998	0,7
4,45	4969	3,98	1938	3,98	619	3,75
4,22	4390	4,22	2248	3,98	565	3,52
4,22	8223	4,22	2726	4,22	846	3,52
4,22	10485	4,22	2468	3,98	889	3,52

4,22	11347	3,98	1574	3,75	1088	2,34
4,22	12873	3,98	2713	3,98	1616	3,28
4,22	15869	4,22	1472	3,98	1244	3,28
4,22	15780	4,22	1406	3,98	964	3,28
4,22	9132	4,22	1105	3,75	1212	3,28
3,98	5154	4,22	1114	3,75	1635	3,28
4,22	4024	3,98	3656	3,75	1374	0,7
4,22	3190	3,98	4575	3,98	2187	0,94
3,98	2556	3,98	2662	3,98	2225	0,94
3,98	2911	3,98	1180	3,75	1205	0,94
3,98	2217	3,98	778	3,75	819	0,94
2,34	1027	0,7	408	0,7	3632	0,47
0,7	1888	1,41	1148	0,7	3048	1,41
0,7	2175	0,94	2021	1,17	2551	0,7
0,7	2254	0,94	3815	2,11	1033	0,47
0,7	2918	1,17	1645	1,41	1494	0,47
0,7	2670	1,17	2125	1,41	2115	0,47
0,47	934	0,47	2096	1,41	1784	0,7
3,75	768	0,47	1976	1,41	1085	1,64
2,81	864	0,47	1175	0,7	711	0,94
0,47	1801	0,94	2021	0,47	762	1,17
0,7	2623	0,47	2626	1,41	1784	1,17
2,81	1861	1,17	2347	1,41	2620	0,7
1,64	737	1,17	1372	0,94	1049	0,7
0,94	1165	1,17	462	0,94	1625	3,05
0,7	984	0,7	1357	0,94	1207	3,05
0,47	210	3,75	351	1,41	167	1,41
3,52	176	3,75	335	1,41	361	1,41
1,41	204	3,75	296	1,41	490	1,41
3,75	325	3,75	312	0,94	165	0,94
0,7	329	3,75	285	0,47	311	1,17
0,7	563	3,75	215	0,47	300	1,17
0,94	917	0,94	195	3,52	200	1,17
0,94	1191	1,17	337	3,52	133	0,7
0,94	942	1,17	414	1,41	147	1,17
0,94	168	1,17	367	0,94	326	4,22
1,41	204	0,94	375	0,7	415	2,34
0,7	184	1,17	167	0,7	654	1,41
1,17	141	0,47	87	0,7	335	1,41
0,94	132	0,47	130	0,47	214	0,94
3,75	193	1,41	144	0,47	183	0,94

riv-6-pow T16	riv-6-df T17	riv-6-pow T17	riv-6-df T18	riv-6-pow T18	riv-6-df T19	riv-6-pow T19
855	0,7	833	1,17	5381	1,41	2107
624	0,94	735	8,67	3365	1,41	1170
710	2,11	1719	1,64	2098	1,41	940
745	2,11	1371	1,41	2978	1,41	2519
1261	2,11	776	8,91	2591	1,17	2451
1848	0,23	644295	0,7	2018	0,94	694
2404	0,47	13671321	0,7	3106	0,94	1127
2081	0,47	28778631	1,17	2570	0,7	1939
1538	0,47	6809628	1,64	2456	2,81	2289
988	0,23	308264	0,94	2822	10,78	4808
1158	0,23	17804	1,41	2931	0,94	2325
1125	0,94	5078	1,41	3213	0,94	1239
2752	0,7	1814	1,17	2446	1,64	460
3448	1,41	2650	0,47	1754	4,45	275
1378	1,41	3547	1,17	1191	0,94	297
281	12,89	1106	0,7	1775	1,41	703
682	12,89	877	0,7	2973	3,75	729
797	1,17	693	0,7	1701	1,17	740
1828	1,17	1470	12,19	677	1,17	786
1563	1,17	1268	0,94	472	11,48	702
1823	1,17	945	1,17	902	11,48	705
2093	1,17	481	1,17	1249	1,41	601
2672	3,98	506	1,17	641	1,41	454
2906	3,98	351	12,19	600	0,94	412
2076	1,64	590	1,41	660	1,17	864
1451	13,36	788	11,02	698	1,17	2873
729	12,89	949	11,25	459	1,41	6111
682	12,42	1019	11,95	400	1,17	6838
968	12,42	1333	12,42	555	1,17	3810
1030	12,19	1616	12,42	674	1,17	4279
1164	1,17	1227	0,7	3081	1,17	796
1158	1,17	1810	0,7	3961	1,41	687
891	0,94	781	1,41	3773	2,11	382
2479	0,94	759	1,41	2063	2,11	402
3522	1,17	1275	0,7	4304	1,17	580
1773	1,17	1345	0,7	7724	0,94	483
1337	1,88	1121	1,88	11441	1,64	514
1847	1,88	684	0,94	3800	0,7	496
1838	1,64	546	0,94	919	0,7	971
1429	1,64	1162	0,94	2219	0,7	646
803	0,94	3946	0,94	6331	0,94	767
1419	1,17	6294	0,94	4749	1,17	853
2130	1,41	2960	0,7	1564	1,17	901
3008	1,17	1100	1,41	1130	0,7	801
2708	0,94	1433	0,7	663	0,94	683
910	0,94	680	2,58	1003	2,34	273
525	11,25	983	2,34	511	2,34	221
396	11,25	1987	2,11	812	0,47	126
269	11,25	2342	2,34	668	1,17	237

329	11,25	1422	2,34	825	1,17	380
641	3,52	1111	2,34	710	3,98	169
669	3,52	1139	1,17	683	2,11	242
252	11,25	557	1,17	442	2,11	424
406	11,25	261	2,58	277	4,22	652
469	10,78	386	2,58	415	3,52	252
374	3,75	730	2,58	618	3,52	373
678	3,75	687	2,58	475	3,28	255
1009	3,75	511	0,94	549	3,75	300
1380	2,34	460	2,58	352	3,75	799
1983	2,58	610	2,34	252	3,75	936
1057	1,17	2644	0,94	4525	0,7	2091
966	1,17	1026	0,94	1255	0,7	2752
788	1,64	467	1,17	635	0,7	3276
2067	0,94	758	1,17	1314	1,41	2198
1979	0,94	3162	1,17	1928	1,41	6485
919	0,94	3558	1,17	1859	1,41	8060
555	0,94	1479	0,94	1043	1,17	2224
465	0,94	910	0,94	1197	1,17	1131
487	0,94	1162	0,94	1954	1,17	1234
1822	0,94	1654	0,94	3384	0,94	1493
1763	0,94	2149	0,94	5539	2,34	1338
1640	0,47	2243	0,94	4442	1,41	1546
1200	0,7	3622	0,94	1646	1,17	1738
2119	0,7	2089	3,28	1104	0,94	4499
2434	1,17	3530	0,7	941	0,94	5534
168	0,94	71	1,41	217	1,17	210
326	1,88	74	1,17	600	1,88	193
209	4,69	132	0,94	838	1,17	136
198	0,7	173	0,7	300	3,75	241
260	0,7	165	0,7	85	3,75	289
193	0,23	169	1,88	87	1,17	247
86	0,7	415	0,47	87	1,17	180
63	0,94	707	0,7	103	0,94	223
127	0,94	464	0,7	53	0,7	514
98	11,02	97	11,02	49	0,94	344
92	1,64	67	11,02	87	0,94	133
39	1,88	77	11,02	84	0,94	113
68	0,94	374	0,94	176	3,75	87
84	0,7	749	1,41	243	1,64	116
141	0,47	199	1,41	265	1,41	217

riv-6-df T20	riv-6-pow T20	riv-6-df T21	riv-6-pow T21	riv-6-df T22	riv-6-pow T22	riv-6-df T23
3,52	676	0,94	2877	1,17	1083	13,83
3,52	1084	0,94	3420	1,17	845	13,83
3,52	842	1,64	1569	1,41	468	1,41
1,64	598	1,41	785	1,41	385	1,88
0,94	532	1,41	521	0,94	440	
0,7	1674	2,81	600	0,94	586	
0,7	2753	2,81	437	1,17	719	
0,7	1429	3,28	558	1,17	1225	
0,94	1583	3,28	688	0,47	1061	
0,94	915	0,47	518	8,44	1353	
0,94	1498	2,81	493	7,27	4191	
0,94	3091	0,94	557	1,41	10367	
0,94	1057	1,17	1104	1,17	14319	
10,08	187	1,17	1782	1,17	6319	
0,94	414	1,17	1863	13,59	3024	
0,94	6757	12,19	350	1,17	917	9,38
0,94	2125	12,42	310	1,17	440	0,94
0,7	794	2,11	568	11,72	228	0,94
12,66	403	0,94	574	11,25	479	0,7
1,17	507	0,94	569	11,25	291	
0,7	414	1,17	904	1,17	91	
10,78	610	1,17	713	13,59	118	
10,78	356	11,95	617	1,41	133	
12,19	963	11,95	877	1,41	336	
12,19	1276	11,95	732	1,17	266	
11,95	952	1,41	432	10,55	354	
11,95	693	1,41	445	10,08	303	
0,94	550	12,19	564	9,61	319	
0,94	639	11,95	441	9,61	502	
11,95	483	0,94	522	9,61	314	
0,94	1291	2,81	741	1,64	187	0,94
0,94	904	0,7	351	1,41	269	0,94
2,58	457	0,47	274	1,41	342	1,41
0,47	331	0,94	202	1,41	556	0,94
0,47	459	1,64	378	0,94	807	0,94
1,41	351	1,88	290	0,94	410	
1,17	2217	1,88	305	0,7	298	
0,94	5769	0,7	402	1,41	309	
0,94	3208	0,94	399	1,41	302	
0,94	808	0,94	252	1,41	169	
1,41	607	1,17	333	0,7	329	
1,17	1294	1,41	314	0,7	518	
1,17	2760	0,7	488	0,47	385	
0,94	3425	0,7	610	1,17	393	
0,94	1720	1,64	215	1,17	419	
3,52	625	3,75	771	0,94	4860	0,7
1,17	743	3,75	293	0,94	6539	3,98
1,17	835	4,45	287	0,94	3644	3,98
0,94	1867	1,41	330	13,36	884	4,22

4,22	1239	1,41	682	13,36	984	0,94
4,22	468	0,94	842	1,41	1361	3,75
1,17	544	3,98	1044	0,94	2189	3,98
4,45	339	3,98	1746	0,94	2232	
3,28	205	3,75	1797	0,94	973	
3,52	589	3,75	897	3,75	1086	
3,75	1048	3,75	380	3,75	836	
3,75	555	3,98	208	3,75	889	
3,52	582	3,98	349	3,98	2434	
3,52	777	3,98	831	3,75	1284	
3,52	778	3,75	1698	0,94	3825	
0,47	1957	1,17	974	1,64	1524	1,41
0,47	2187	1,88	474	3,05	897	1,41
0,94	1190	0,47	916	0,94	1307	1,41
1,41	799	0,7	1256	1,64	1027	1,41
0,7	1590	0,7	592	0,7	1105	1,17
0,7	2456	0,7	568	0,7	2570	2,11
0,94	3160	1,64	1948	0,94	1902	
1,41	3581	1,64	1670	1,41	588	
1,17	1529	0,94	982	2,11	325	
1,17	3753	0,7	2611	0,47	599	
0,94	3044	1,41	2302	0,7	1565	
0,7	2333	1,41	3024	0,7	3153	
0,7	2626	0,94	2424	0,94	1943	
0,47	1996	1,64	3069	0,94	1802	
0,47	1324	1,64	4972	1,41	1832	
1,41	308	1,17	117	1,17	153	1,41
1,17	431	1,17	145	11,72	182	0,94
1,17	440	1,17	201	11,72	104	0,94
0,7	443	1,41	533	11,02	102	
0,7	631	1,41	1273	0,94	104	
1,17	401	1,41	641	0,47	98	
1,17	1377	1,17	307	3,52	66	
0,94	648	0,94	219	0,94	134	
0,7	417	0,94	87	1,17	317	
1,88	207	0,7	182	1,17	334	
0,7	146	0,7	233	0,7	94	
0,94	179	0,94	468	3,28	145	
0,7	86	0,94	737	3,05	212	
1,41	122	0,7	491	0,7	746	
1,41	157	0,7	225	0,94	1249	

riv-6-pow T23		Animal No	Bas-riv-12-df
3174	Rivastigmin 12 mg	311 b	3,52
2811			0,94
1012			1,41
3165			3,52
			3,52
			3,52
			0,94
			0,94
			3,52
			3,52
			3,52
			0,94
			2,11
			2,81
			3,05
140		312 b	1,41
267			1,41
708			1,64
607			1,64
			1,88
			0,94
			0,7
			0,7
			3,28
			3,28
			3,52
			0,7
			0,7
			1,17
			3,75
410		313 b	1,17
269			1,17
548			1,17
360			0,94
801			2,58
			2,81
			0,47
			0,47
			0,47
			0,47
			0,94
			0,94
			1,17
			2,58
			2,81
5180		314 b	1,17
2613			3,28
2228			3,52
1686			3,52

2628		0,94
2785		1,17
2603		1,17
		1,17
		1,17
		0,47
		0,7
		0,47
		1,41
		3,05
		3,28
1956	315 b	3,28
1212		3,28
1325		3,05
1551		3,05
2178		3,98
1079		3,28
		2,81
		7,5
		7,73
		7,73
		1,17
		0,47
		0,23
		3,28
		3,05
544	316 b	0,23
347		0,7
259		0,7
		0,47
		0,47
		0,7
		0,7
		0,7
		0,7
		0,94
		1,17
		0,94
		0,94
		1,41
		1,41

Bas-riv-12-pow	riv-12-df T1	riv-12-pow T1	riv-12-df T2	riv-12-pow T2	riv-12-df T3	riv-12-pow T3
9039	3,05	7385	3,52	1702	0,94	822
11977	3,28	7067	3,52	1337	3,52	880
12664	3,28	7593	3,52	532	3,52	2746
10513	3,28	5962	3,75	1153	3,52	2727
9285	3,28	6238	3,52	3139	3,75	2022
7053	3,52	4896	3,52	5153	3,75	1174
8290	3,52	8751	3,52	4534	3,52	2074
3077	3,52	11886	3,52	1672	3,75	1749
4855	3,28	11636	3,52	668	3,75	696
8504	3,28	7976	1,88	712	3,75	606
5093	3,28	3238	3,75	702	3,75	593
2202	3,52	3355	3,52	707	3,75	504
2238	3,28	3849	3,52	659	3,75	418
3727	3,28	2061	3,28	485	3,75	247
7385	3,52	1585	0,94	598	3,75	157
753	3,75	288	3,28	3038	3,28	4511
461	3,75	354	3,28	3714	3,28	4247
249	0,94	218	3,28	4698	3,28	3811
226	3,05	1359	3,28	4213	3,28	3361
126	3,05	1803	3,28	3380	3,28	2706
177	3,05	1910	3,28	3414	3,28	2072
677	3,05	2329	3,28	4143	3,28	2572
937	3,05	1938	3,52	5488	3,28	3279
627	3,05	1609	3,52	6501	3,28	4106
846	3,05	1628	3,52	6860	3,28	4969
531	3,05	1931	3,52	5339	3,28	5057
573	3,28	2653	3,52	3601	3,28	4666
812	3,05	2720	3,28	1652	3,28	4277
357	3,28	2524	3,28	2776	3,28	5395
288	3,28	3873	3,28	4291	3,28	5786
10204	2,81	998	2,81	790	0,7	885
9307	3,05	996	0,7	978	0,94	464
11849	3,05	643	0,7	2833	2,81	598
3996	2,81	920	0,7	6663	2,81	901
634	2,81	834	0,7	3392	2,81	786
276	2,81	1316	2,81	755	2,81	720
284	2,81	2124	0,7	558	2,81	579
603	2,58	2217	0,7	1034	2,81	252
1808	2,58	1377	0,7	756	2,81	311
1531	2,81	698	2,81	483	0,7	237
1806	2,81	341	2,81	823	0,94	475
3871	2,81	630	1,17	1630	2,81	476
2196	2,81	1661	1,17	1116	2,81	341
863	2,81	2329	2,81	713	2,81	462
1013	2,81	1762	2,81	611	1,41	844
934	3,28	485	7,73	96	0,47	69
437	3,52	491	3,52	97	0,47	102
214	3,75	280	2,34	140	1,17	97
210	3,75	103	2,11	117	1,17	175

250	1,64	170	1,17	127	7,03	122
549	1,64	157	1,17	117	2,81	137
716	1,41	180	1,64	83	3,75	197
524	3,75	215	1,64	131	0,7	328
366	1,17	141	1,17	191	3,52	408
425	2,81	158	1,17	493	3,52	377
821	0,7	233	1,17	302	3,52	232
755	0,94	222	0,47	177	3,75	179
551	0,7	306	0,47	596	3,52	131
444	0,7	238	1,64	681	3,52	203
485	0,7	70	1,64	215	3,52	355
3201	3,05	768	3,05	1498	3,98	2351
4362	3,05	1162	2,81	353	3,98	2299
2899	3,05	762	8,44	328	3,98	2409
631	3,28	1642	3,05	406	3,98	2033
311	3,05	1915	3,05	532	3,98	1542
566	3,05	1643	3,05	519	3,98	1406
403	3,05	983	3,05	579	3,98	1649
593	3,05	810	3,28	313	3,98	1978
647	3,05	1348	8,44	579	3,98	1760
475	3,05	1622	8,44	595	3,98	1532
1048	2,81	952	2,81	1343	3,98	1120
3522	2,81	813	2,81	2174	3,28	2069
2437	3,28	706	3,98	3742	3,98	2042
1204	3,28	776	3,98	3194	3,98	2330
768	3,05	1909	3,98	2448	3,75	2259
12918	1,41	2334	0,94	7261	1,17	5175
70020	1,41	2956	0,7	3571	0,7	3157
175292	1,41	3085	1,41	3330	0,7	3454
72235	1,17	1972	0,47	4163	0,7	4042
18270	0,94	4672	0,7	6089	0,7	1435
28327	0,94	3044	0,94	2620	1,17	467
32503	0,94	1598	0,7	566	3,52	401
10535	0,7	2144	3,52	295	1,17	432
1711	1,17	1667	3,98	530	1,41	1005
784	0,94	2277	2,81	1050	0,7	4206
3379	1,41	9248	2,81	1334	0,7	6073
9172	1,17	59184	0,94	1781	0,47	2516
6542	0,94	100584	0,94	4720	1,17	2904
2198	0,94	99010	0,94	8617	1,17	3507
2334	0,94	48561	1,17	6120	0,94	2411

riv-12-df T4	riv-12-pow T4	riv-12-df T5	riv-12-pow T5	riv-12-df T6	riv-12-pow T6	riv-12-df T7
3,05	300	3,52	3021	3,28	1806	3,28
2,81	291	3,28	1994	3,28	1303	3,28
3,28	386	3,28	660	3,52	773	3,28
3,52	995	3,75	449	0,94	459	3,28
3,75	1114	3,75	781	3,05	363	3,05
3,75	843	3,75	1553	3,05	391	2,81
3,75	748	3,52	665	3,05	173	3,05
3,05	935	3,28	475	0,7	329	3,05
3,75	1229	3,28	913	0,7	523	0,94
3,75	1115	3,52	1232	3,28	682	3,05
1,17	1138	3,52	1278	3,28	1270	3,05
1,17	1123	3,28	2028	3,28	1383	3,05
1,17	429	3,52	3220	3,28	956	0,7
3,05	1790	3,52	3224	3,28	1213	3,28
3,05	2169	3,52	1475	3,28	1602	3,28
3,52	4457	3,52	5586	3,52	2649	1,17
3,28	3328	3,75	5220	3,52	2563	3,75
3,52	5481	3,75	5903	3,75	2375	3,75
3,52	8542	3,75	8655	3,75	1971	3,75
3,52	7196	3,75	11786	3,52	2410	1,17
3,52	6114	3,75	6703	3,75	4662	1,17
3,52	5317	3,75	2523	3,75	7215	0,94
3,52	4483	3,75	2064	3,75	4446	3,98
3,52	2634	3,52	2298	3,75	3178	3,98
3,52	1922	3,52	1721	3,75	2684	3,75
3,52	2552	3,75	877	3,52	1235	3,75
3,52	3415	3,75	855	0,94	555	0,23
3,52	3890	3,75	854	3,05	637	3,52
3,52	3616	3,52	1143	3,52	779	3,52
3,52	4430	3,52	2573	3,52	806	3,52
3,05	536	0,94	1015	0,94	1384	3,28
3,05	269	0,94	783	0,94	819	3,28
3,28	298	0,94	929	2,34	200	0,7
0,94	259	0,94	287	0,94	258	0,94
0,7	359	4,45	81	0,94	916	1,64
2,58	356	1,17	487	0,7	790	1,88
2,34	269	1,41	2710	0,7	223	2,58
2,34	153	1,41	1770	1,64	169	0,7
1,17	225	1,17	428	1,64	393	0,47
1,17	542	1,64	738	1,64	718	1,41
1,17	525	0,7	346	0,94	864	0,94
1,17	488	3,28	212	0,94	1001	1,17
1,17	623	0,7	281	0,7	435	1,17
0,94	714	1,64	358	1,88	311	1,41
0,94	1418	1,17	494	1,17	402	1,17
3,52	372	3,75	207	3,98	2309	3,98
1,17	310	3,75	310	3,98	2399	3,98
1,17	599	3,98	455	3,98	2125	3,98
1,41	701	3,98	802	3,98	1670	3,98

1,41	415	3,98	940	3,98	1290	3,98
1,41	211	3,98	853	3,98	1458	3,98
3,52	299	3,98	1081	3,98	2081	3,98
1,17	420	3,98	1374	3,98	3435	3,98
1,17	375	3,98	1587	3,98	4906	3,98
3,52	369	3,98	1444	3,98	3869	3,75
3,52	347	3,98	1140	3,98	2110	3,75
7,27	249	3,98	1197	3,98	1082	3,98
3,75	257	3,98	1482	3,98	650	3,98
3,75	313	3,98	1602	3,98	718	3,98
3,28	234	3,98	1833	3,98	1012	7,73
3,75	3065	3,75	4106	3,28	2746	1,41
3,98	3013	3,75	2935	3,52	3298	1,41
3,98	3752	4,92	1438	1,64	2229	3,98
3,98	3706	3,28	3617	3,98	932	3,98
3,98	3377	1,88	3873	3,75	684	3,75
3,98	3039	1,64	8369	3,75	549	1,17
0,94	4855	1,64	5873	4,22	251	1,41
0,94	10754	3,98	3308	3,98	785	3,52
1,17	7170	3,75	3846	3,98	1002	3,52
1,41	7922	3,75	4494	3,98	920	3,52
1,41	9663	3,75	2956	3,98	749	3,28
1,41	9209	3,52	1985	3,75	589	3,98
1,41	7962	3,75	1739	3,52	1640	3,98
1,64	4822	3,98	1639	3,98	1994	3,75
3,98	4379	3,98	1607	3,98	1867	3,75
0,94	809	0,47	485	1,41	801	1,17
1,17	799	1,17	170	1,17	1393	3,52
0,94	386	1,17	283	0,94	373	3,28
3,05	288	0,7	598	3,28	259	0,94
1,17	543	0,7	1319	0,94	343	0,94
0,7	595	0,7	642	1,64	354	3,28
1,17	347	0,94	345	1,64	447	3,52
1,17	158	2,58	505	2,81	356	1,17
3,05	122	0,94	593	3,28	581	1,17
1,17	580	0,94	502	1,17	406	3,52
1,17	2028	3,05	725	0,7	1634	1,41
0,7	1790	3,05	854	0,7	3097	1,41
0,7	1968	3,05	536	0,7	1144	1,41
1,17	1597	3,05	249	3,52	472	1,17
1,41	924	3,05	86	3,28	867	1,17

riv-12-pow T7	riv-12-df T8	riv-12-pow T8	riv-12-df T9	riv-12-pow T9	riv-12-df T10	riv-12-pow T10
2096	3,05	667	0,47	535	3,75	1358
2448	0,94	559	3,05	685	3,75	1171
3866	0,94	1406	2,81	1727	3,75	1562
4034	1,17	1073	2,81	1339	3,75	1773
2151	3,28	613	3,75	1194	3,75	2176
537	3,28	803	3,75	1213	1,41	4571
540	0,94	929	3,05	1524	1,41	4308
576	0,94	759	3,05	2622	0,7	2772
248	3,52	1293	3,05	3135	3,75	2879
515	3,52	2015	3,05	2157	3,75	3976
768	3,52	1811	3,52	1190	3,75	4196
482	3,52	1237	3,75	1567	3,75	3020
260	3,52	988	3,75	2447	3,75	2328
434	3,52	843	3,75	3114	3,75	2167
484	3,52	355	3,75	2185	3,75	2127
1006	3,52	511	3,52	1933	2,81	614
1190	3,52	838	3,52	2508	1,64	526
1476	3,52	913	3,52	2439	1,64	1001
2020	3,52	673	3,52	2194	1,64	445
1949	3,52	349	3,28	2084	2,11	281
3619	0,94	423	3,28	1787	1,88	812
1553	0,94	385	3,28	801	1,64	1182
2002	3,28	702	1,41	494	1,41	816
1876	3,52	1023	3,05	1309	1,41	903
1053	3,52	1463	3,05	2064	1,64	702
282	3,52	1549	3,28	1585	1,64	467
292	3,28	1685	3,28	1739	0,47	289
432	3,28	1931	3,28	2047	0,7	329
387	3,28	2111	3,28	1707	0,94	509
330	3,28	2174	3,52	537	3,28	1341
298	0,47	340	0,94	374	1,41	1068
235	1,17	275	0,7	304	1,41	1669
271	0,94	369	3,05	199	1,17	1388
287	0,7	276	2,81	223	0,94	707
364	0,7	228	1,41	168	1,17	699
467	1,64	159	1,17	1125	1,17	766
436	1,17	247	1,17	24683	1,41	867
307	1,17	288	1,17	143900	1,41	756
129	0,94	771	1,64	211939	0,7	369
133	0,7	1400	0,7	126067	3,28	206
223	0,7	447	1,17	178090	3,28	181
832	3,05	138	0,94	298043	1,41	461
652	0,94	236	0,7	71208	1,41	837
291	3,52	550	0,47	4440	1,17	538
180	3,75	485	1,64	223	0,94	95
1767	7,97	435	3,52	683	3,52	312
2656	7,73	438	3,52	809	1,41	278
3022	7,73	361	0,94	888	0,7	434
3498	0,94	326	0,94	799	0,94	520

3826	0,47	214	1,41	460	0,94	422
4069	3,75	290	3,52	355	3,52	332
4623	3,75	250	5,63	237	3,52	384
4071	3,75	315	3,05	357	3,28	551
2516	3,75	461	11,72	186	3,28	335
1305	3,75	436	11,95	381	0,94	237
1157	3,75	347	11,95	416	1,17	262
709	3,52	386	11,95	327	3,28	331
645	3,52	704	3,05	204	3,28	440
299	3,52	843	3,05	270	3,28	336
326	3,52	730	3,28	234	3,28	299
1219	3,98	645	1,17	8514	1,17	4301
1144	2,81	1872	1,17	6789	0,94	4502
783	1,64	7825	2,11	4029	3,52	3904
1206	1,64	5333	3,52	3995	1,17	5363
676	3,05	2430	3,52	6975	1,17	7711
853	3,05	2819	3,52	9220	1,17	9653
625	3,05	2028	3,52	7666	1,17	16199
864	3,05	1309	3,52	2404	1,17	16833
1616	3,75	727	3,52	2286	0,94	11973
1379	3,75	1085	3,52	5170	0,94	15963
661	3,52	548	3,52	5837	1,17	22039
637	1,17	839	3,52	6847	1,17	21802
547	3,52	1033	3,52	6027	1,17	18149
552	1,41	1000	3,75	4531	0,94	18446
665	1,17	3015	1,17	3383	1,17	33166
1012	0,7	1851	3,75	953	3,52	1370
768	0,94	1996	3,75	1494	0,94	2218
596	0,94	1619	3,75	1484	0,94	4240
507	0,94	739	3,75	1749	3,52	2261
769	0,7	1066	3,75	1683	3,52	1967
526	0,7	3796	3,52	1491	3,52	1600
408	0,7	3349	3,52	1648	3,52	1076
778	0,94	2874	3,52	1374	3,52	678
660	0,94	3105	3,52	1512	3,28	1478
363	0,94	2329	3,52	1665	3,28	2648
1021	1,17	1153	3,52	1704	3,28	2292
1459	1,41	1055	3,52	1994	3,28	523
1139	3,52	795	3,52	1876	2,81	403
1881	0,7	753	3,52	1243	1,41	275
673	0,7	893	3,52	1238	1,17	214

riv-12-df T11	riv-12-pow T11
3,75	1995
3,75	2050
3,75	3219
3,75	3644
1,64	2532
3,05	4215
3,05	3021
3,28	1284
3,28	624
3,28	527
3,75	372
3,05	640
3,75	676
3,75	1128
0,94	1178
3,28	1849
1,41	2204
3,52	2548
3,52	1408
3,28	1301
1,17	1390
1,41	2481
1,41	4351
1,41	5101
1,41	3938
1,17	1660
1,64	1973
3,52	2051
3,52	3139
1,17	5922
3,28	45
0,47	60
3,75	111
1,17	163
1,17	249
1,17	325
0,94	363
0,7	250
3,52	193
1,17	370
0,94	448
1,17	257
0,94	140
0,94	195
1,17	307
3,28	289
1,88	358
1,64	340
1,17	517

1,17	493
3,28	241
3,52	460
3,52	435
3,98	237
0,94	331
0,94	433
3,52	735
3,52	469
3,52	234
3,52	412
1,17	26815
1,17	12685
3,75	8509
3,75	6169
3,52	8241
1,17	9899
1,17	11451
1,17	8429
1,17	12374
1,17	42345
1,17	47154
1,17	15676
1,17	14035
1,17	29501
1,17	19573
1,41	219
1,64	355
2,81	300
0,47	118
1,17	205
1,17	546
1,41	552
0,7	365
0,7	598
0,7	743
1,17	769
0,7	899
2,58	576
0,7	681
1,88	674
